# Long-life Li/polysulphide batteries with high sulphur loading enabled by lightweight three-dimensional nitrogen/sulphur-codoped graphene sponge

**DOI:** 10.1038/ncomms8760

**Published:** 2015-07-17

**Authors:** Guangmin Zhou, Eunsu Paek, Gyeong S. Hwang, Arumugam Manthiram

**Affiliations:** 1 Materials Science and Engineering Program & Texas Materials Institute, The University of Texas at Austin, 204 East Dean Keeton Street, Mail Stop: C2200, Austin, Texas 78712, USA.; 2 McKetta Department of Chemical Engineering, The University of Texas at Austin, 204 East Dean Keeton Street, Mail Stop: C2200, Austin, Texas 78712, USA.

## Abstract

Lithium–sulphur batteries with a high theoretical energy density are regarded as promising energy storage devices for electric vehicles and large-scale electricity storage. However, the low active material utilization, low sulphur loading and poor cycling stability restrict their practical applications. Herein, we present an effective strategy to obtain Li/polysulphide batteries with high-energy density and long-cyclic life using three-dimensional nitrogen/sulphur codoped graphene sponge electrodes. The nitrogen/sulphur codoped graphene sponge electrode provides enough space for a high sulphur loading, facilitates fast charge transfer and better immobilization of polysulphide ions. The hetero-doped nitrogen/sulphur sites are demonstrated to show strong binding energy and be capable of anchoring polysulphides based on first-principles calculations. As a result, a high specific capacity of 1,200 mAh g^−1^ at 0.2C rate, a high-rate capacity of 430 mAh g^−1^ at 2C rate and excellent cycling stability for 500 cycles with ∼0.078% capacity decay per cycle are achieved.

## Abstract (web summary)

There is intensive research underway into the cathode development of lithium–sulphur batteries. Here, the authors report a lithium–sulphur battery using nitrogen/sulphur codoped graphene structure which displays excellent electrochemical performance with high sulphur loading.

## Graphical abstract

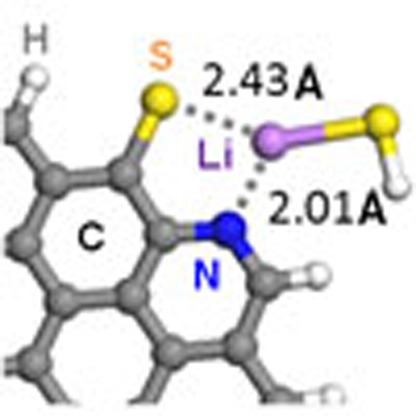

As energy storage devices, lithium-ion batteries (LIBs) play a dominant role in portable electronic devices due to their high performance compared with other battery systems^1^,^2^. However, the increased demand for electric vehicles and large-scale smart grid stringently requires batteries with high-energy density, low cost and long cycle life^3^. The current main challenge is the capacity mismatch between the cathode and anode, which makes LIB approach its theoretical energy density limits^4^,^5^. The relatively lagged progress on cathodes becomes a barrier in further improving the energy density of LIBs, which has triggered the exploration of new electrochemical systems, such as the Lithium–sulphur (Li–S) batteries with a high theoretical energy density of 2,600 Wh kg^−1^ (refs 6, 7). Despite the considerable advantages of Li–S battery, several problems prevent it from practical applications: (1) the insulating characteristic of sulphur and its discharge products (Li_2_S), leading to a low utilization of active material; (2) large volumetric expansion/shrinkage (80%) during discharge/charge, resulting in an instability of the electrode structure; (3) the soluble intermediates (Li_2_S_*x*_, 3≤*x*≤8) in the organic liquid electrolyte during the cycle process bring about the polysulphide ‘shuttle effect', which leads to irreversible capacity loss and corrosion on the lithium-metal anode^4^,^8^,^9^.

Much effort has been devoted to solving these issues: improving the electrical conductivity of the sulphur electrode by integrating with carbon materials or conductive polymers^8^,^9^,^10^,^11^,^12^,^13^,^14^; designing yolk-shell structure with internal void space to accommodate the volume expansion of sulphur^15^,^16^; using novel electrolyte additives such as P_2_S_5_ or Cs^+^ to suppress the polysulphide shuttle and passivate the lithium-metal surface^17^,^18^; and modifying the surface chemistry of the hosts to prevent the shuttle effect of the soluble polysulphides between the cathode and the anode, enabling better cycle performance^19^,^20^,^21^,^22^. However, the areal loading of sulphur electrode in most of the reported work is <2.0 mg cm^−2^ and the sulphur content in the electrode is <70 wt. %, which are not enough to satisfy the demands for high-energy density batteries^5^,^12^,^23^,^24^,^25^. Generally, conventional electrode requires the use of inactive materials such as conductive agents, metallic current collectors and binders, which will also offset the high-energy density advantage of Li–S batteries.

Moreover, flat current collectors face the challenge of increasing the electrode thickness due to the easy delamination of active materials and limitation in lithium diffusion kinetics. Therefore, three-dimensional (3D) battery electrode design has recently been explored as an effective way to enhance the energy per surface area of batteries^26^,^27^. For example, Miao *et al*.^28^ synthesized 3D carbon fibre cloth current collectors to host sulphur for achieving high sulphur loading and high areal capacity in Li–S batteries; Wu's group^29^ impregnated sulphur into a 3D graphene framework to increase the sulphur areal mass loading in the composite electrodes; Yuan *et al*.^30^ adopted a bottom-up strategy to fabricate 3D hierarchical free-standing carbon nanotube-S paper electrodes to improve the sulphur content; and Cheng's group^31^ reported flexible 3D graphene foams as the current collector to accommodate large amount of sulphur with high areal capacity. However, with the increase of sulphur loading, the internal sulphur is not easily accessible to the electrolytes and hard to be used rapidly, which leads to the phenomenon of ‘sulphur activation' with lower capacity in the initial few cycles^28^,^29^,^31^. Therefore, a simpler and more effective way to enable high sulphur utilization, especially when sulphur loading is high, is still challenging while worth exploring.

Instead of adopting solid sulphur as the precursor followed by melt-diffusion or liquid infiltration methods, the dissolved lithium polysulphide systems have recently been proved to make active materials distribute more uniformly, improve sulphur utilization, alleviate electrode structure variation and facilitate the kinetics of the Li–S redox reaction^5^,^7^,^32^,^33^,^34^,^35^. Taking the above discussion into consideration, we present the use of a lightweight, porous nitrogen- and sulphur- (abbreviated as N, S) codoped 3D graphene sponge as the additive/binder-free electrode structure to accommodate large amounts of dissolved lithium polysulphides. The active materials could reach as high as 4.6 mg cm^−2^ when the graphene sponge is employed as a 3D current collector. The interconnected graphene network facilitates fast electron and ion transfer. The sulphur and/or nitrogen doping could facilitate fast charge transfer, increase the affinity between the polysulphide species and the carbon-based framework, facilitate better immobilization of the polysulphide ions, and promote the Li_2_S/polysulphide/S reversible conversion to improve the electrochemical performance of Li–S batteries^19^,^21^,^22^,^36^. The combination of physical adsorption of lithium polysulphides onto porous graphene and the chemical binding of polysulphides to N and S sites in graphene suppresses sulphur loss during the discharge/charge processes, enabling a high specific capacity of 1,200 mAh g^−1^ at 0.2C rate, a high-rate capacity of 430 mAh g^−1^ at 2C rate and excellent cycling stability for 500 cycles with nearly 100% Coulombic efficiency for the N,S-codoped graphene electrode.

## Results

### Synthesis and characterization of graphene-based sponges

**Figure 1:**
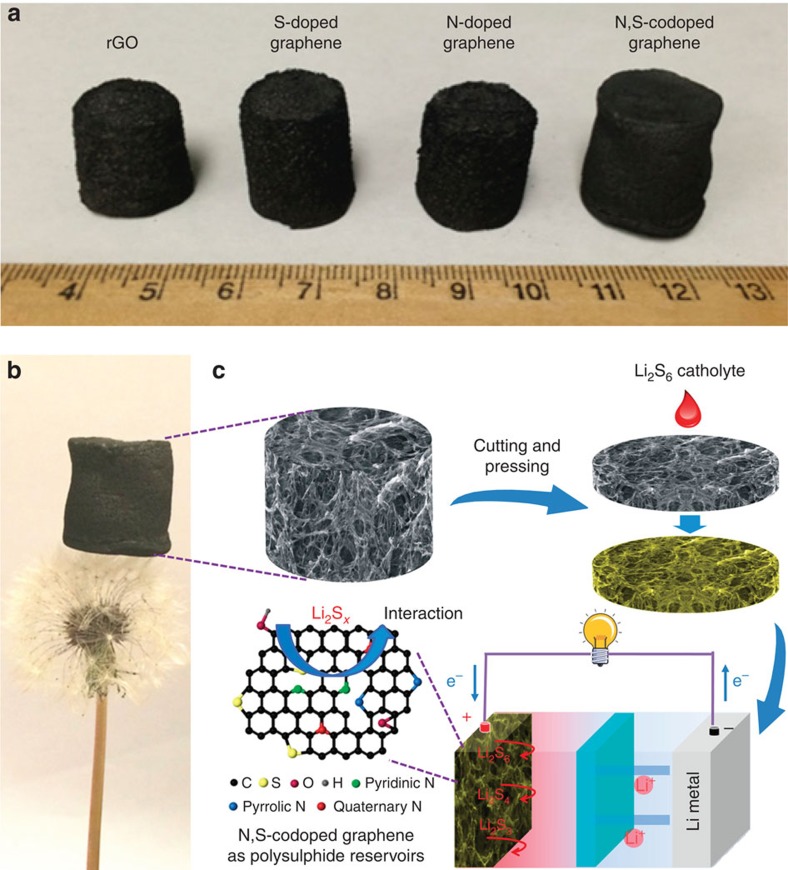
Photographs of graphene sponges and schematic model of the assembled cell. (**a**) Photographs of the rGO, S-doped graphene, N-doped graphene and N,S-codoped graphene sponges after the hydrothermal reaction and freeze-drying. (**b**) A lightweight N,S-codoped graphene sponge standing on a dandelion. (**c**) Illustration of the formation process of the N,S-codoped graphene electrode and schematic of the fabrication of a Li/dissolved polysulphide cell with N,S-codoped graphene electrode after adding polysulphide catholyte.

Figure 1a demonstrates the typical photographs of the as-prepared reduced graphene oxide (rGO), S-doped graphene, N-doped graphene and N,S-codoped graphene sponges obtained by hydrothermal reaction using, respectively, GO, GO/Na_2_S, GO/urea and GO/thiourea as precursors followed by freeze-drying. Na_2_S, urea and thiourea were employed as sulphur, nitrogen and nitrogen/sulphur sources, as well as reducing agents for GO during the hydrothermal assembly process. After freeze-drying, the morphology of the samples was retained and no obvious shrinkage/expansion is observed. The size and shape of the graphene-based sponges can be adjusted by controlling the concentration of GO, reaction temperature and the volume/shape of the used autoclaves. The freeze-dried N,S-codoped graphene sponge is light and can stand stably on the top of a dandelion without deforming it at all (Fig. 1b), which makes it a good candidate as a lightweight 3D current collector. It can be cut and pressed into slices for direct use as a host for dissolved lithium polysulphides without metal current collectors, binders and conductive additives, and the sulphur/nitrogen doping in graphene is suggested to interact with lithium polysulphide/Li_2_S to improve the cycle stability of Li–S batteries, as illustrated in Fig. 1c.

**Figure 2:**
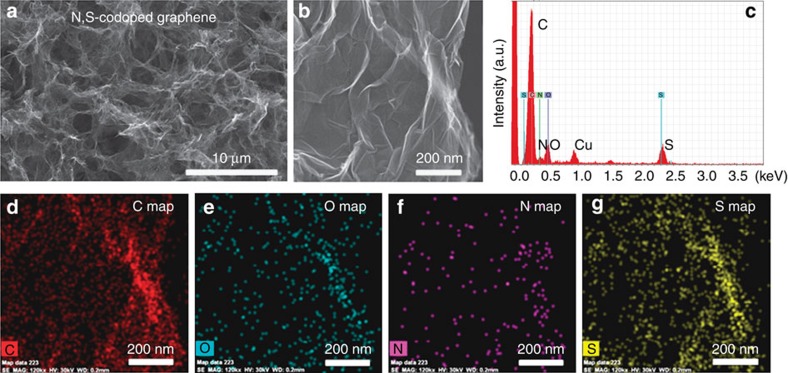
Morphology and microstructure of the N,S-codoped graphene sponge. (**a**) SEM image of the N,S-codoped graphene sponge. (**b**) High-magnification SEM image of the N,S-codoped graphene sponge. (**c**) EDS spectrum of the N,S-codoped graphene. (**d**) Carbon, (**e**) oxygen, (**f**) nitrogen and (**g**) sulphur elemental mappings of the N,S-codoped graphene in **b**.

Figure 2a and Supplementary Figure 1 show the typical scanning electron microscopy (SEM) images of the graphene-based sponges. The alkaline solution environment, when using Na_2_S and urea as doping sources, is not beneficial for the formation of intact nitrogen- or sulphur-doped graphene sponges in a one-pot hydrothermal reaction due to the increased electrostatic repulsion between the rGO sheets^37^,^38^, so the synthesis of rGO sponge followed by sulphur and nitrogen doping strategy is adopted. Therefore, the rGO, S-doped graphene and N-doped graphene sponges show quite similar morphologies (Supplementary Fig. 1a–c), displaying a relatively loose structure with large pores of tens of micrometres formed by linked graphene sheets. In contrast, the N,S-codoped graphene sponge cross-links to form a 3D interconnected network structure with rich pores in a smaller size of 2–8 μm (Fig. 2a). The macropores in the 3D hierarchical network structure allow efficient ion diffusion/mass transfer and could also be used as the buffering/absorption reservoir of polysulphides^39^,^40^. Scanning transmission EM (STEM) and high-magnification SEM characterizations reveal that the resultant N,S-codoped graphene sponge consists of wrinkled graphene sheets (Fig. 2b and Supplementary Fig. 2). N_2_ isothermal adsorption–desorption analysis (Supplementary Fig. 3, Supplementary Table 1) reveals that the Brunauer–Emmmett–Teller specific surface areas of N,S-codoped graphene, N-doped graphene and S-doped graphene sponges are, respectively, 171.4 m^2^ g^−1^, 136.7 m^2^ g^−1^ and 133.5 m^2^ g^−1^, which are slightly higher than that of the rGO sponge (108.7 m^2^ g^−1^). The N_2_ adsorption–desorption isotherms of all the graphene-based sponges show a main adsorption at a relative pressure >0.9 with a small hysteresis loop (Supplementary Fig. 3a), indicating the predominant meso/macropore structure, consistent with the pore size distributions (Supplementary Fig. 3b) and SEM/STEM observation (Fig. 2a, Supplementary Figs 1 and 2a). Energy-dispersive X-ray spectroscopy (EDS) reveals the presence of C, O, Cu (from Cu grid), N and S in the N,S-codoped graphene sponge (Fig. 2c), indicating the successfully incorporated N,S atoms into the carbon framework. The EDS elemental mapping further confirms the existence and homogeneous distribution of C, O, N and S on the surface of N,S-codoped graphene (Fig. 2d–g). These functional groups are beneficial to restrict the polysulphides from escaping to the electrolyte, which will be discussed below.

The transformation from GO to the final graphene-based sponges was examined by X-ray diffraction (XRD) and Raman spectroscopy (Supplementary Fig. 4). GO shows the typical diffraction peak at ∼11° (ref. 41). After the hydrothermal reaction, this peak disappears while a broad diffraction peak emerges between 18° and 28° in the XRD patterns for rGO, S-doped graphene, N-doped graphene and N,S-codoped graphene sponges, demonstrating the reduction of GO (Supplementary Fig. 4a). Moreover, this peak for the N,S-codoped graphene sponge is sharper and shifted to higher angles compared with that of rGO, S-doped graphene and N-doped graphene sponges, suggesting the more efficient reduction process by N,S-doping. Raman spectra further provide additional evidence of the doping and reduction processes (Supplementary Fig. 4b). Two prominent peaks are observed at 1,365 cm^−1^ and 1,590 cm^−1^, corresponding to the characteristic D and G bands of carbon materials^41^, respectively. Generally, the intensity ratio of the D band to G band (*I*_D_/*I*_G_) is used to evaluate the degree of defects in carbon materials, and a higher ratio indicates an increased amount of defects that is proportional to the extent of reduction^41^. Compared with GO (*I*_D_/*I*_G_=0.93), the *I*_D_/*I*_G_ ratio increases to 0.99, 1.03, 1.07 and 1.16 for rGO, S-doped graphene, N-doped graphene and N,S-codoped graphene sponges, respectively, suggesting the removal of oxygen-containing groups and the insertion of heteroatoms into the graphene network with more structural defects^42^. The increased *I*_D_/*I*_G_ ratio is indicative of the enhanced reduction, which is in agreement with the electrical conductivity tests of these sponges (Supplementary Fig. 5).

**Figure 3:**
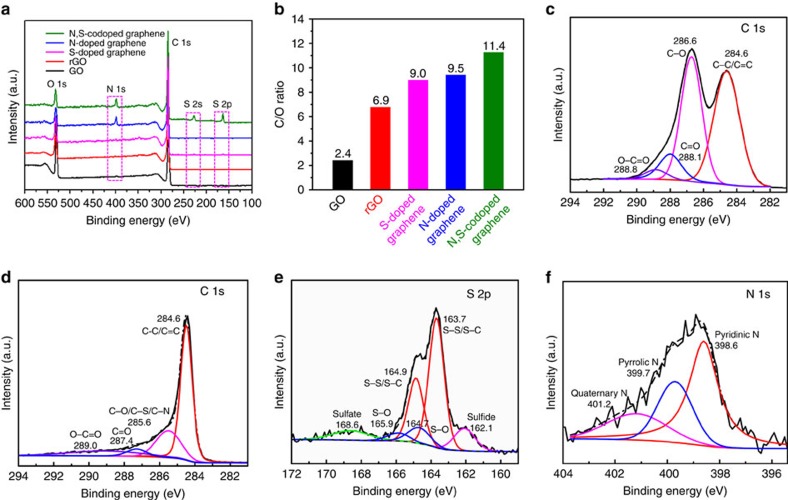
Surface composition analysis. (**a**) XPS spectra of the surface chemical composition of GO, rGO, S-doped graphene, N-doped graphene and N,S-codoped graphene. (**b**) Relationship of the C/O ratio in the GO, rGO, S-doped graphene, N-doped graphene and N,S-codoped graphene. C 1s XPS spectra of the (**c**) GO and (**d**) N,S-codoped graphene. (**e**) S 2p XPS spectrum of the N,S-codoped graphene. (**f**) N 1s XPS spectrum of the N,S-codoped graphene.

The surface chemical composition and functional groups of the GO, rGO, S-doped graphene, N-doped graphene and N,S-codoped graphene were identified by X-ray photoelectron spectroscopy (XPS), as shown in Fig. 3. For GO and rGO, only C 1 s and O 1 s signals were detected in the XPS survey spectra. After nitrogen and nitrogen/sulphur doping, a new peak located at ∼400.5 eV is observed in both the N-doped graphene and N,S-codoped graphene, corresponding to N 1 s^43^; two other peaks appearing at 164.5 and 228.2 eV for S-doped graphene and N,S-codoped graphene are attributed to S 2p and S 2 s (Fig. 3a), respectively, suggesting the efficient S-doping in graphene. The elemental contents in these samples are summarized in Supplementary Table 2. The nitrogen contents in the N-doped graphene and N,S-codoped graphene reach, respectively, 5.1 at.% and 5.4 at.%, and the sulphur contents are 0.6 at.% and 3.9 at.%, respectively, in S-doped graphene and N,S-codoped graphene, which further confirm that the S and/or N are incorporated into the carbon framework. GO shows a C/O ratio of 2.4 and this value increases to 6.9 for rGO after the hydrothermal reaction, implying the partial reduction of GO (Fig. 3b). After the S, N- and N/S-doping, the C/O ratio further increases to, respectively, 9.0, 9.5 and 11.4, indicating a more efficient reduction during the doping process, which is consistent with the XRD and Raman results. The deconvoluted C 1 s spectra of GO show four peaks at 284.6 eV, 286.6 eV, 288.1 eV and 288.8 eV (Fig. 3c), respectively, corresponding to C–C/C=C, C–O, C=O and O–C=O^44^. After the N,S-doping, these oxygen-containing groups decrease significantly, and the peak intensity corresponding to the sp^2^ carbon increases and becomes narrower (Fig. 3d), indicating the removal of oxygen-containing functional groups and the possible formation of C-S/C-N bonds during the reduction process^22^,^45^. The S 2p spectra of the N,S-codoped graphene can be deconvoluted into six peaks (Fig. 3e), which correspond to, respectively, sulphide at 162.1 eV, S–S/S–C bonds at 163.7 and 164.9 eV, S–O species at 164.7 and 165.9 eV, and sulfate species at 168.6 eV (refs 20, 36, 45, 46, 47). In the N1s spectrum (Fig. 3f), the three different peaks at 398.6 eV, 399.7 eV and 401.2 eV are ascribed to, respectively, pyridinic N, pyrrolic N and quaternary N^21^,^43^. These functional groups are suggested to contribute to improving the affinity and binding energy of the nonpolar carbon atoms with polar polysulphides/Li_2_S, thus significantly enhancing the cycle stability and rate capability of Li–S batteries^19^,^20^,^21^,^36^.

### Adsorption capabilities and electrochemical performance

The polysulphide adsorption capabilities of these graphene-based sponges were investigated with ultraviolet–visible absorption spectroscopy. Pure Li_2_S_6_ solution was used as a reference, the rGO, S-doped graphene, N-doped graphene and N,S-codoped graphene sponges were immersed into the Li_2_S_6_ solution for 2 h and the concentration variation of Li_2_S_6_ solution was analysed. The typical peaks of the polysulphide solution located at 260, 280, 300 and 340 nm (Supplementary Fig. 6) are attributed to the S_6_^2−^ species^48^,^49^,^50^. After the absorption for 2 h, it can be clearly seen that the intensity decreases in these characteristic absorption peaks and the N,S-codoped graphene sponge exhibits the best adsorption capability compared with the rGO, S-doped graphene and N-doped graphene sponges, demonstrating the improvement in affinity and adsorption between the S_6_^2−^ species and the graphene after the N,S functionalization.

**Figure 4:**
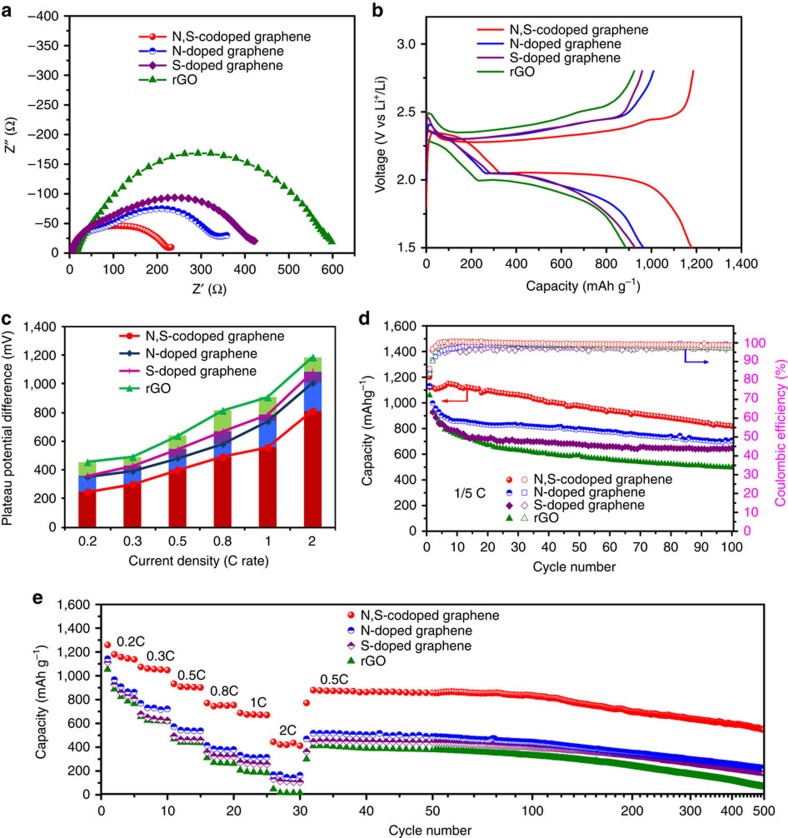
Electrochemical performance of Li/polysulphide batteries with different graphene electrodes. (**a**) Nyquist plots of the rGO, S-doped graphene, N-doped graphene and N,S-codoped graphene electrodes before cycling from 1 MHz to 100 mHz at room temperature. (**b**) The second galvanostatic charge/discharge profiles of the rGO, S-doped graphene, N-doped graphene and N,S-codoped graphene electrodes at 0.2C rate within a potential window of 1.5–2.8 V versus Li^+^/Li^0^. (**c**) Comparison of the potential difference between the charge and discharge plateaus at different current densities. (**d**) Cycling performance and Coulombic efficiency of the Li polysulphide batteries with the rGO, S-doped graphene, N-doped graphene and N,S-codoped graphene electrodes at 0.2C rate for 100 cycles. (**e**) Rate performance of the rGO, S-doped graphene, N-doped graphene and N,S-codoped graphene electrodes at different current densities and long-term cycle stability of the corresponding electrodes at 0.5C for 500 cycles after the high-current-density test.

To show whether the doped heteroatoms of S and/or N on the graphene have a positive effect on the performance of Li–S batteries, these graphene sponges were cut into slices, compressed and directly used as free-standing electrodes for a series of electrochemical measurements. Different from two-dimensional graphene materials, 3D interconnected porous graphene sponges can provide multidimensional electron transport pathways and easily accessible active sites. Therefore, electrochemical impedance spectroscopy was used to analyse the kinetics of the electrochemical reactions. Figure 4a shows the Nyquist plots of the graphene-based sponge electrodes at the open-circuit voltage before cycling, and the equivalent circuit is shown in Supplementary Fig. 7. The high frequency intercept at the real axis corresponds to the internal impedance (*R*_e_), and the semicircles located in the higher and lower frequency regions, respectively, correspond to the surface film resistance (*R*_f_) and the charge-transfer resistance (*R*_ct_) of the batteries^51^. The other elements in the equivalent circuit include a constant phase element (CPE) about the double-layer capacitance, Warburg impedance (*Z*_w_) and space charge capacitance (CPE′)^52^. From the plots, it can be seen that the rGO electrode has the largest internal resistance (13.7 Ω), while this resistance in the S-doped graphene (5.5 Ω), N-doped graphene (4.3 Ω) and N,S-codoped graphene (3.7 Ω) electrodes are lower, which are in accordance with the results of the electrode electrical conductivity (Supplementary Fig. 5). The *R*_ct_ of the N,S-codoped graphene electrode is much smaller than those of the rGO, S-doped graphene and N-doped graphene electrodes (Supplementary Table 3), which could be attributed to the interconnected porous graphene network and doped N/S atoms, facilitating the charge transfer for surface reactions^21^,^22^,^36^. Figure 4b demonstrates the galvanostatic charge/discharge profiles of the Li/dissolved polysulphide batteries with the rGO, S-doped graphene, N-doped graphene and N,S-codoped graphene electrodes at 0.2C rate (1C=1,675 mA g^−1^). Compared with the rGO, S-doped graphene and N-doped graphene electrodes, the discharge/charge profiles of the N,S-codoped graphene electrode have an obvious higher discharge plateau at ∼2.32 V (reduction of sulphur to long-chain lithium polysulphides) and a longer plateau at ∼2.10 V (formation of short-chain lithium polysulphides) with corresponding charge plateaus at ∼2.30 and ∼2.45 V (transformation from Li_2_S_2_/Li_2_S to long-chain lithium polysulphides and then to sulphur)^53^. These plateaus are longer and flatter with lower polarization and are well-retained even at higher current densities of 0.3–2C rates between the charge/discharge processes when compared with those of rGO, S-doped graphene and N-doped graphene electrodes (Fig. 4c and Supplementary Fig. 8), suggesting better redox reaction kinetics with good reversibility. For example, the overpotential is 809 mV in the N,S-codoped graphene electrode at a high rate of 2C, much lower than that of rGO electrode (1,185 mV), S-doped graphene (1,080 mV) and N-doped graphene electrode (1,010 mV).

The cycle performances of these electrodes were first tested at a small current density of 0.2C rate, as shown in Fig. 4d. The N,S-codoped graphene electrode delivers an initial discharge capacity of 1,200 mAh g^−1^, and the capacity is retained at 822 mAh g^−1^ after 100 cycles with nearly 100% Coulombic efficiency, which is much higher than that of the rGO, S-doped graphene and N-doped graphene electrodes with values of 495 mAh g^−1^, 646 mAh g^−1^ and 705 mAh g^−1^, respectively. Lithium nitrate (LiNO_3_) is added to promote the formation of a stable passivation film, and the capacity decay at the initial few cycles is partially due to the LiNO_3_ decomposition and the formation of a SEI film on the lithium anode surface^54^,^55^. To further elucidate the electrochemical reactivity of doped sulphur in the graphene framework, the blank galvanostatic charge/discharge profiles and the cyclic stability of N,S-codoped graphene are shown in Supplementary Fig. 9. It can be seen that the first discharge capacity is 219 mAh g^−1^, and the voltage plateau at ∼1.6 V is attributed to the reduction of LiNO_3_ on the carbon and lithium surface^55^. The capacity remains to be only 46 mAh g^−1^ after 100 cycles, demonstrating its little contribution to the overall capacity. To demonstrate the rate capabilities of these electrodes, the current density was changed from 0.2 to 2C rate, as shown in Fig. 4e. The average discharge capacities for the N,S-codoped graphene electrode at 0.2C, 0.5C, 1C and 2C rates are, respectively, 1,157 mAh g^−1^, 912 mAh g^−1^, 675 mAh g^−1^ and 430 mAh g^−1^, indicating a high charge/discharge capability. However, the rGO electrode shows almost no capacity at 2C rate, demonstrating poor rate performance. When the current density was reduced back to 0.5C rate, the discharge capacity can be recovered to 878 mAh g^−1^ for the N,S-codoped graphene electrode, indicating that the electrode structure remains stable after the high-current-density test. Even after the rate capability test, the N,S-codoped graphene electrode still shows excellent long-cyclic performance with higher capacity (550 mAh g^−1^) and retention (63%) compared with the rGO (66 mAh g^−1^, 16%), S-doped graphene (190 mAh g^−1^, 42%) and N-doped graphene (221 mAh g^−1^, 43%) electrodes at a current density of 0.5C rate up to 500 cycles. The capacity decay is only 0.078% per cycle for the N,S-codoped graphene electrode, much better than those of the rGO, S-doped graphene and N-doped graphene electrodes with decay rates of 0.179%, 0.124% and 0.121% per cycle, respectively. These results imply that the nitrogen- and sulphur-doped 3D porous graphene sponge could not only facilitate fast electron/ion transfer, but also interact strongly with lithium polysulphides to significantly enhance the rate performance and long-term cyclic stability.

**Figure 5:**
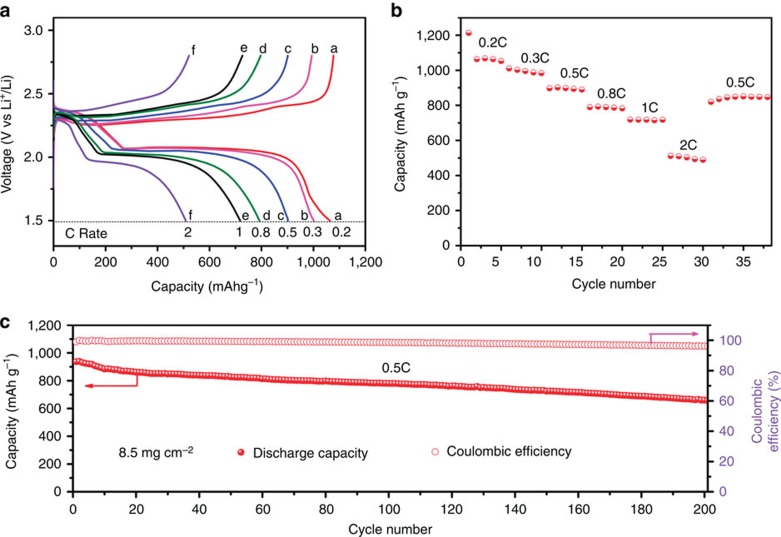
Electrochemical measurement of N,S-codoped graphene electrode with graphene-coated separator. (**a**) Galvanostatic charge/discharge profiles of the N,S-codoped graphene electrode with graphene-coated separator at various rates within a potential window of 1.5–2.8 V versus Li^+^/Li^0^. (**b**) Rate performance of the N,S-codoped graphene electrode with graphene-coated separator at different current densities. (**c**) Cycling performance and Coulombic efficiency of the Li polysulphide batteries with the N,S-codoped graphene electrodes and graphene-coated separator at 0.5C rate for 200 cycles.

To further improve the energy density of the whole battery system and satisfy the demands of high-energy batteries, the active material loading was increased from 4.6 to 8.5 mg cm^−2^ by adding more Li_2_S_6_ catholyte into the N,S-codoped graphene electrode with the help of a graphene-coated separator, which has been confirmed as a very useful approach to obtain high-performance Li–S batteries^56^. From the charge/discharge curves of the N,S-codoped graphene electrodes with graphene-coated separator (Fig. 5a), it is obviously observed that two discharge/charge plateaus are well-retained even at a high rate of 2C, indicating that the reaction dynamic is not affected by high sulphur loading. Owing to the uniform distribution of liquid-phase active materials, the polysulphides could be utilized effectively, thereby avoiding the phenomenon of ‘sulphur activation'^28^,^29^,^31^. The unique structure enables the battery to deliver a high capacity of 1,070 mAh g^−1^ at 0.2C rate, and the reversible discharge capacity could reach *ca.* 500 mAh g^−1^ at a large current density of 2C rate (Fig. 5b). In addition, a long-term cyclic test at 0.5C rate was carried out and the initial specific capacity is 925 mAh g^−1^, which stabilizes ∼670 mAh g^−1^ after 200 cycles (Fig. 5c). The advantages of such electrode configuration design includes the following: (i) the graphene sponge functions as a 3D current collector to accommodate a large amount of active material, and the internal graphene layer further improves the polysulphide utilization due to the increased overall electrical conductivity of the system; (ii) the graphene layers effectively trap the dissolved lithium polysulphide and provide more active sites for Li_2_S deposition; (iii) the 3D graphene network and graphene layer can accommodate the volume change of the active material during cycling; and (iv) the chemical binding between the sulphur/nitrogen heteroatoms and lithium polysulphide/Li_2_S reduces the active material loss, realizing long cycle life and high-energy/power density Li–S batteries.

## Discussion

To understand the structure and surface modification in improving the performance of N,S-codoped graphene electrode, the cells were disassembled inside the glove box and the surface morphology of the graphene-based sponge cathodes and the lithium metallic anodes after 100 cycles were observed by SEM. It can be seen that a thick layer of lithium sulphide (Li_2_S) deposition is present on the surface of rGO and the corresponding metallic lithium surface is rough with many coarse lithium agglomerations (Supplementary Figs 10a and 11a), suggesting a serious parasitic reaction between dissolved lithium polysulphides and metallic lithium anode during cycling. This can also be confirmed from the corresponding sulphur elemental mapping characterization and EDS compositional analysis (Supplementary Fig. 11b, Supplementary Table 4). In contrast, less deposition is seen on the S-doped graphene and N-doped graphene electrodes (Supplementary Fig. 10b,c), and no obvious aggregations of sulphur species are observed on the N,S-codoped graphene electrode (Supplementary Fig. 10d), showing less dissolution loss of polysulphides into the electrolyte and their re-deposition on the S-doped, N-doped and N,S-codoped graphene electrodes. Correspondingly, the lithium surface of S-doped graphene, N-doped graphene and N,S-codoped graphene electrodes is also smoother (Supplementary Fig. 11c,e,g) with respect to the rGO electrode, showing that less side reactions have occurred on the surface of metallic lithium, thereby alleviating the lithium surface corrosion and contributing to the improved cycling performance (Supplementary Fig. 11d,f,h and Supplementary Table 4).

**Figure 6:**
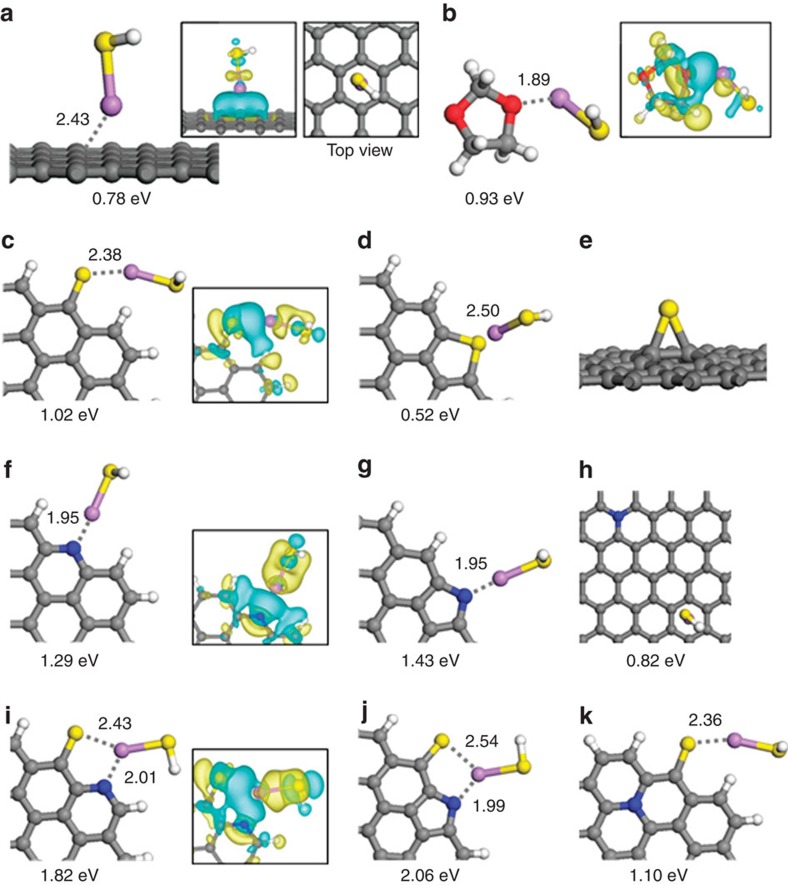
Theoretical calculations. Optimized configurations for the binding of LiSH to (**a**) pristine graphene, (**b**) 1,3-dioxolane, (**c–e**) S-doped graphene, (**f–h**) N-doped graphene and (**i–k**) N,S-codoped graphene. Charge density difference isosurfaces are shown in the insets; the blue and yellow colours indicate the regions of charge gain and loss (of ±0.001 e per bohr^3^), respectively. Grey, white, blue, yellow, purple and red balls represent C, H, N, S, Li and O atoms, respectively. LiSH binding energies (in eV) and selected bond distances (in Å) are also indicated.

To better understand the doping effect, density functional theory (DFT) calculations were performed to examine how the binding strength of the Li–S end of linear lithium polysulphides (Li_2_S_*x*_) is influenced by the incorporation of N and/or S atoms into the graphene lattice. Here we used a small LiSH molecule to model the Li–S end. This model is simple but should be sufficient enough to demonstrate the influence of doping particularly on the binding interaction of the terminal Li^+^ cation with a doped graphene sheet, although lithium polysulphides may also exist as complex clusters^57^,^58^,^59^. To confirm whether the H-terminated S will influence the predicted binding strength of the terminal Li^+^, we also considered Li_2_S adsorption at a few selected dopant sites, as shown in Supplementary Fig. 12. There are no significant binding energy differences between Li_2_S and LiSH, implying that it would be reasonable and feasible to use LiSH for the purpose of supporting our experimental observations. For a reference, we first considered the binding of LiSH to a pristine graphene sheet and a 1,3-dioxolane (DOL) molecule, which is widely used for the electrolyte in a Li–S cell. As shown in Fig. 6a, the Li of LiSH is preferentially located at the hollow site above the centre of a hexagon ring with a predicted binding energy of *E*_b_=0.78 eV, in good agreement with the previous DFT results^57^. Here the *E*_b_ is given by *E*_LiSH_+*E*_Gr_−*E*_LiSH/Gr_, where *E*_LiSH_, *E*_Gr_ and *E*_LiSH/Gr_ represent the total energies of an isolated LiSH, pristine (or doped) graphene and LiSH adsorbed graphene, respectively. Our calculations also predict that the Li of LiSH can be strongly bound to the O site of DOL with *E*_b_=0.93 eV (Fig. 6b); the larger *E*_b_ than that for the graphene case (0.78 eV) may suggest that lithium polysulphides would dissolve into the DOL-based electrolyte instead of adsorbing on the graphene surface, consistent with the existing experimental observations^10^,^47^.

Next, we examined the interaction of LiSH with doped graphene. Figure 6 shows the adsorption configurations of LiSH at highly probable binding sites identified from our DFT calculations; (Fig. 6c–e) S doped, (Fig. 6f–h) N doped and (Fig. 6i–k) N,S codoped. Compared with the case of pristine graphene, the *E*_b_ is predicted to increase by 0.24 eV at the thionic S site (Fig. 6c), but decrease by 0.26 eV at the thiophenic S site (Fig. 6d). We also considered the interaction of LiSH with S placed on the basal plane of graphene (Fig. 6e), but the weakly bound S turns out to easily desorb off the graphene surface by forming LiS_2_H. LiSH is also found to strongly interact with pyridinic and pyrrolic N at the edge of graphene (Fig. 6f,g); the predicted *E*_b_ of 1.29 eV and 1.43 eV, respectively, are substantially greater than that of the pristine graphene case (0.78 eV). On the other hand, the quaternary N doping (Fig. 6h) appears to insignificantly affect the LiSH binding strength, although the electron-rich surface due to electron donation from N likely leads to the increase in *E*_b_ to a certain extent. Overall, our results are consistent with the previously reported DFT results^19^,^21^,^22^. Looking at N,S-codoped graphene, our DFT calculations predict that thionic S can exist adjacent to pyridinic or pyrrolic N, as shown in Fig. 6i,j; the total energy of the codoped graphene tends to increase (decrease) with the S–N distance in the case of pyridinic (pyrrolic) N, but only marginally (<0.05 eV). When thionic S and pyridinic (pyrrolic) N are located nearby, the binding strength of LiSH is predicted to increase significantly, yielding *E*_b_=1.82 (2.06) eV, as compared with the separately doped cases (Fig. 6f,g). In addition, we find that LiSH is likely to interact more strongly with thionic S in the quaternary N-doped graphene (Fig. 6k), in comparison to the case of single doped graphene (Fig. 6c).

N and S atoms could be incorporated into graphene in various configurations, yielding a wide range of *E*_b_; a few additional cases are presented in Supplementary Fig. 13. Nonetheless, our DFT results clearly demonstrate that the coexistence of N and S can significantly enhance the binding of lithium polysulphides, when compared with the undoped or single N/S-doping cases. In addition, according to our calculations, thionic S and pyridinic (or pyrrolic) N could exist adjacent to each other at graphene edges and vacancies, which may in turn lead to an increase in the amount of dopants, when N and S are codoped as compared with the cases of single N (or S)-doping. Overall, our theoretical findings regarding the synergistic effect of N,S-codoping well-support our experimental observations.

In summary, we have built a strong bound interface between graphene and soluble lithium polysulphides by N,S-codoping to optimize the electrochemical performance towards stable and high-energy density Li–S batteries. The N,S-codoped graphene conductive framework provides high electrical conductivity, strong adsorption abilities for polysulphides and rapid on-transport channels, and works as a 3D scaffold to accommodate high active material loading. As a result, the N,S-codoped graphene electrode with a high sulphur loading of 4.6 mg cm^−2^ exhibits fast reaction dynamics, reduced polarization and stabilized cycling performance with only 0.078% capacity decay per cycle up to 500 cycles. In addition, the sulphur loading could be further increased to 8.5 mg cm^−2^ using the 3D N,S-codoped graphene current collector with the help of a graphene-coated separator. The high loading polysulphides could be effectively utilized with high reversibility and excellent stability. This work demonstrates the great potential of using 3D current collector and surface chemical modifications for high-energy density, long-life energy storage devices.

## Methods

### Synthesis of GO

GO was synthesized using natural graphite flakes by a modified Hummers' method^60^. The concentration of the GO suspension obtained was 2.6 mg ml^−1^, which was determined by freeze-drying the suspension and weighing the dried GO.

### Synthesis of rGO sponge

Hydrothermal assembly and freeze-drying process were combined to fabricate rGO sponge. In a typical procedure, 50 ml of GO suspension was transferred into a 100 ml Teflon-lined stainless steel autoclave and hydrothermally treated at 180 °C for 12 h. Then the autoclave was naturally cooled to room temperature, the black rGO hydrogel was washed with distilled water and the wet hydrogel was freeze-dried to obtain the rGO sponge.

### Synthesis of S-doped graphene sponge

About 0.01 mol of Na_2_S was added to 50 ml of distilled water and the above obtained rGO hydrogel was then transferred into the solution, sealed in the autoclave and maintained at 180 °C for 12 h. After that, the S-doped graphene hydrogel was dipped into distilled water and washed several times to remove the residual salts and freeze-dried to obtain the S-doped graphene sponge.

### Synthesis of N-doped graphene sponge

About 0.01 mol of urea was added to 50 ml of distilled water and stirred magnetically for 30 min. The obtained rGO hydrogel was then transferred into the solution and sealed in the autoclave and maintained at 180 °C for 12 h. After that, the N-doped graphene hydrogel was dipped into distilled water and washed several times to remove the residual urea and freeze-dried to obtain the N-doped graphene sponge.

### Synthesis of N,S-codoped graphene sponge

In brief, 50 ml of the GO aqueous dispersion and 0.01 mol of thiourea were mixed and the mixture was stirred for 30 min and sealed in a 100 ml Teflon-lined stainless steel autoclave for hydrothermal reaction at 180 °C for 12 h. Then the black hydrogel was washed with distilled water several times to remove the residual thiourea and freeze-dried to obtain the N,S-codoped graphene sponge.

### Materials characterization

SEM observations were carried out on a FEI Quanta 650 SEM operated at 20 kV. STEM was performed with a Hitachi S-5500 SEM, and EDS was used for collecting elemental signals and mapping. XRD patterns were collected with a Philips X-ray diffractometer with Cu Kα radiation (*λ*=0.154056, nm) between 10° and 70° at a scan rate of 0.04° s^−1^. Raman spectra were collected with a 488 nm laser under ambient conditions with a WITEC Alpha300 S micro-Raman system at room temperature. XPS analysis was performed on a Kratos Analytical spectrometer at room temperature with monochromatic AlKα (1,486.6 eV) radiation. Ultraviolet–visible absorption spectroscopy analysis was performed to evaluate the polysulphide adsorption capability of rGO, S-doped graphene, N-doped graphene and N,S-codoped graphene sponges. About 4 mg each of rGO, S-doped graphene, N-doped graphene and N,S-codoped graphene sponges were separately added into four sealed vials of Li_2_S_6_ solution (4 ml each, 0.1 mmol l^−1^), and the Li_2_S_6_ solution without adding anything was used as a control. After absorption for 2 h, the ultraviolet–visible absorption spectra of these solutions were collected with an Evolution 300 UV–vis spectrophotometer with baseline correction. The electrical conductivity of the electrodes was measured by a standard four-point probe resistivity measurement system (S-302-4, Lucas Labs Resistivity, USA). Three measurements were taken at different positions on the sample, and the average value was taken.

### Preparation of electrolyte and polysulphide catholyte

The blank electrolyte was prepared by dissolving an appropriate amount of lithium trifluoromethanesulfonate (LiCF_3_SO_3_, 98%, Acros Organics, 1 M) and LiNO_3_ (99+%, Acros Organics, 0.1 M) in DME (99+%, Acros Organics) and DOL (99.5%, Acros Organics) (1:1 by volume). The polysulphide catholyte was prepared by chemically reacting sublimed sulphur (99.5%, Fisher Scientific) and an appropriate amount of Li_2_S (99.9%, Acros Organics) in the blank electrolyte to form Li_2_S_6_ (1.0 M) in the solution. The solution was then stirred at 50 °C in an Ar-filled glove box overnight to produce a brownish-red Li_2_S_6_ catholyte solution.

### Electrochemical measurements

The Li/dissolved polysulphide cells (CR2032 coin cells) were assembled in an Ar-filled glove box. The rGO, S-doped graphene, N-doped graphene and N,S-codoped graphene sponges were dried at 120 °C under vacuum overnight before using. These samples were cut, compressed and shaped into rectangular plates to be used as the current collectors with an area of 0.5 cm^2^ and a mass of 0.9–1.4 mg. About 12 μl of Li_2_S_6_ catholyte (1.0 M) was added into each of the rGO, S-doped graphene, N-doped graphene and N,S-codoped graphene electrodes, corresponding to a sulphur loading of 4.6 mg cm^−2^. The Celgard 2500 separator was then placed on top of the electrode, followed by adding 30 μl of the blank electrolyte. Finally, the lithium-metal foil anode was placed on the separator as the anode. An Arbin battery cycler was used to perform the galvanostatic cycling measurements at 1.5–2.8 V (versus Li/Li^+^) at room temperature. The current density set for tests was referred to the mass of sulphur in the cathode and was varied from 0.2C to 2C rate. For the high sulphur loading cathode, 22 μl of Li_2_S_6_ catholyte (1.0 M) was added into the N,S-codoped graphene electrodes, corresponding to a sulphur loading of 8.5 mg cm^−2^. The graphene-coated separator was used to place on the top of the sulphur cathode followed by adding 50 μl of the blank electrolyte. Electrochemical impedance spectroscopy measurements were performed with a Solartron Impedance Analyzer (Solartron 1260A) in the frequency range of 1 MHz to 0.1 Hz with an AC voltage amplitude of 5 mV at the open-circuit potential. For the cycled samples, the cycled cells were disassembled inside an Ar-filled glove box, and the electrodes were rinsed with 1,2-dimethoxyethane solvent to remove the lithium salt and dried inside the glove box at room temperature before analysis.

### Theoretical calculations

The atomic configurations and binding energies reported herein were calculated using DFT within the Perdew–Berke–Ernzerhof generalized gradient approximation (GGA-PBE)^61^, as implemented in the Vienna *Ab initio* Simulation Package (VASP)^62^. We employed the projector augmented wave method to describe the interaction between ion core and valence electrons and a plane-wave basis set with a kinetic energy cutoff of 400 eV. A graphene sheet was modelled using a hexagonal 7 × 7 supercell; we used the GGA-optimized lattice constant of 2.466 Å, which is close to the experimental value of 2.46 Å. Periodic boundary conditions were employed in all three directions with a vacuum gap of 15 Å in the vertical (*z*) direction to avoid interactions between graphene and its periodic images. To model N and/or S dopant atoms at graphene edges and vacancies, we employed a graphene flake consisting of 37C atoms in a periodic simulation box of dimensions 20 × 20 × 15 Å; dangling C bonds were passivated by H atoms. A gamma-centred 2 × 2 × 1 Monkhorst–Pack mesh of k points was used for the Brillouin zone integration. All atoms were fully relaxed until the residual forces on constituent atoms became smaller than 0.02 eV Å^−1^. We used the semi-empirical approach proposed by Grimme^63^ to take into account the vdW interactions within DFT.

## Additional information

**How to cite this article:** Zhou, G. *et al*. Long-life Li/polysulphide batteries with high sulphur loading enabled by lightweight three-dimensional nitrogen/sulphur codoped graphene sponge. *Nat. Commun.* 6:7760 doi: 10.1038/ncomms8760 (2015).

## Supplementary Material

Supplementary Information Supplementary Figures 1-13 and Supplementary Tables 1-4
