# Supplementary material for: Long-life Li/polysulphide batteries with high sulphur loading enabled by lightweight three-dimensional nitrogen/sulphur-codoped graphene sponge
Source: Nat Commun. 2015 Jul 17;6:7760. doi: 10.1038/ncomms8760 (PMC4518288; doi:10.1038/ncomms8760)
Supplement: Supplementary Information — Supplementary Figures 1-13 and Supplementary Tables 1-4 [file ncomms8760-s1.pdf]

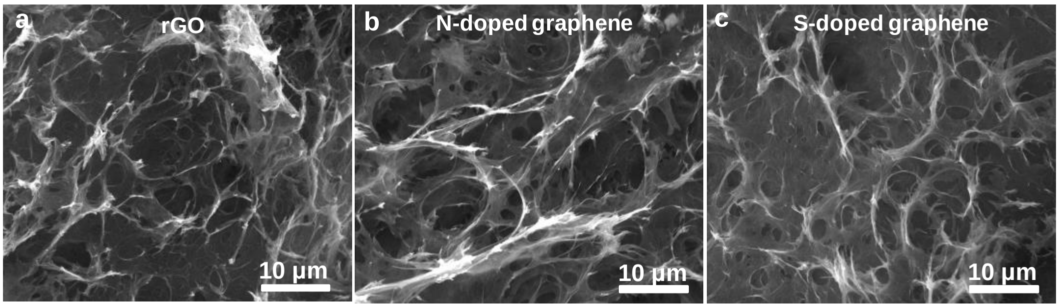

Supplementary Figure 1. SEM images of the (**a**) rGO, (**b**) N-doped graphene, and (**c**) S-doped graphene sponges.

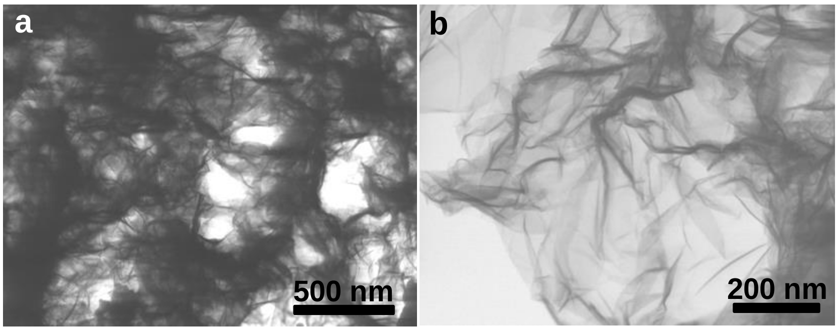

Supplementary Figure 2. (**a, b**) STEM images of the N,S-codoped graphene sponge.

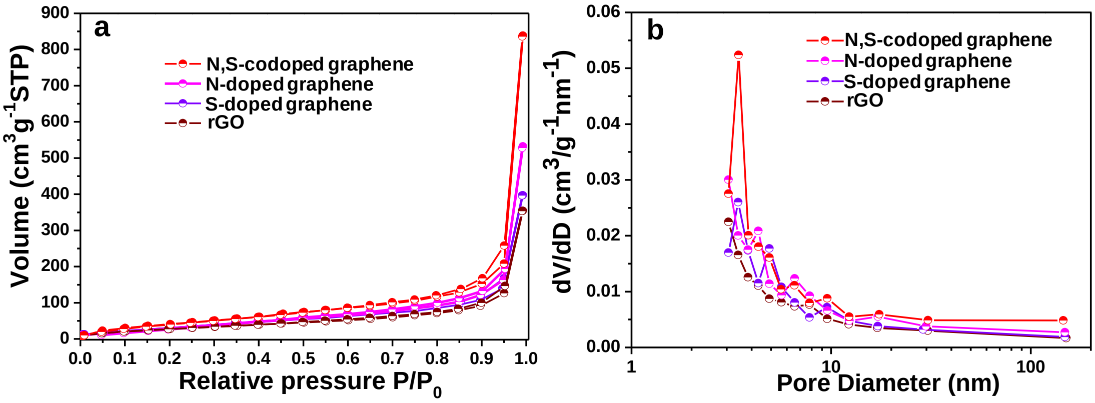

Supplementary Figure 3. (**a**) N_2 adsorption-desorption isotherms of the rGO, S-doped graphene, N-doped graphene, and N,S-codoped graphene. (**b**) Pore-size distributions of the rGO, S-doped graphene, N-doped graphene, and N,S-codoped graphene.

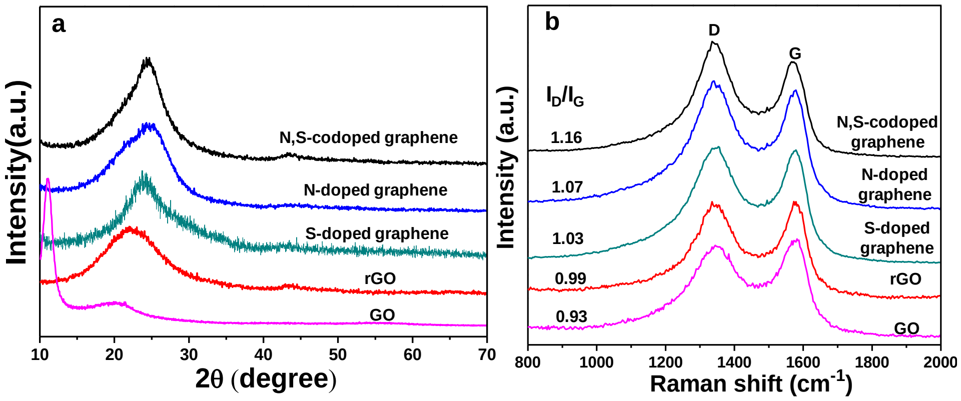

Supplementary Figure 4. (**a**) XRD patterns of the GO, rGO, S-doped graphene, N-doped graphene, and N,S-codoped graphene. (**b**) Raman spectra of the GO, rGO, S-doped graphene, N-doped graphene, and N,S-codoped graphene.

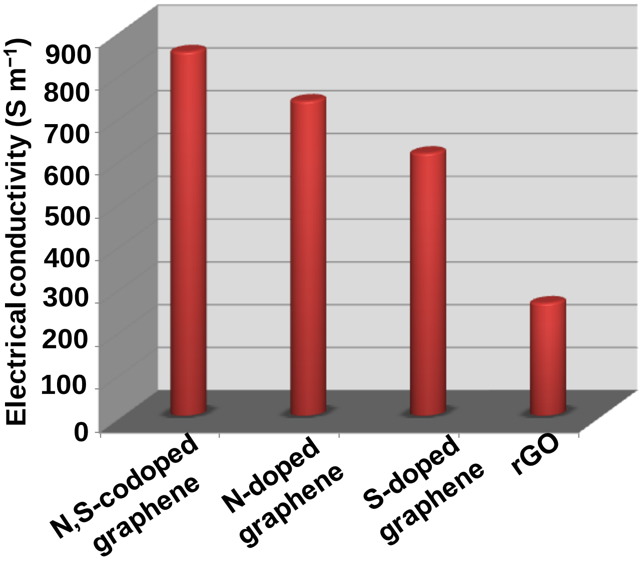

Supplementary Figure 5. Electrical conductivity of the rGO, S-doped graphene, N-doped graphene, and N,S-codoped graphene.

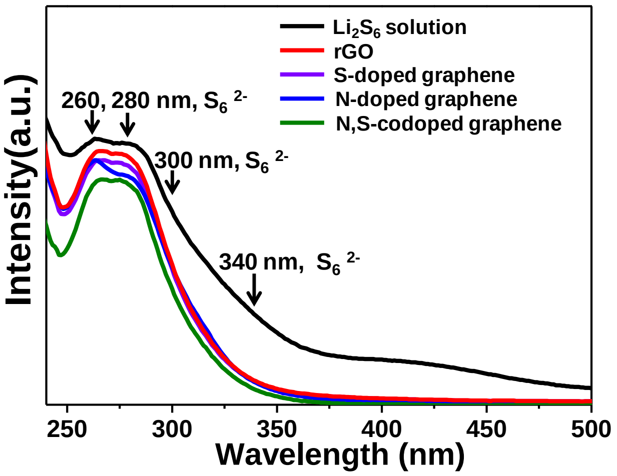

Supplementary Figure 6. UV/Vis absorption spectra of lithium polysulphide (Li_2S_6) solution before and after the addition of rGO, S-doped graphene, N-doped graphene, and N,S-codoped graphene sponges.

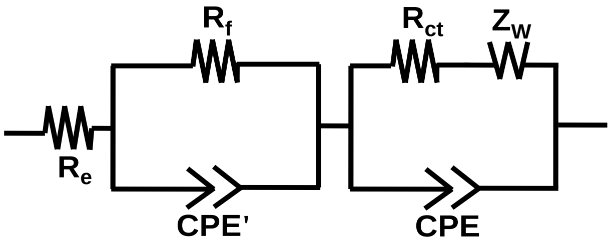

Supplementary Figure 7. Equivalent circuit used for fitting the impedance spectra.

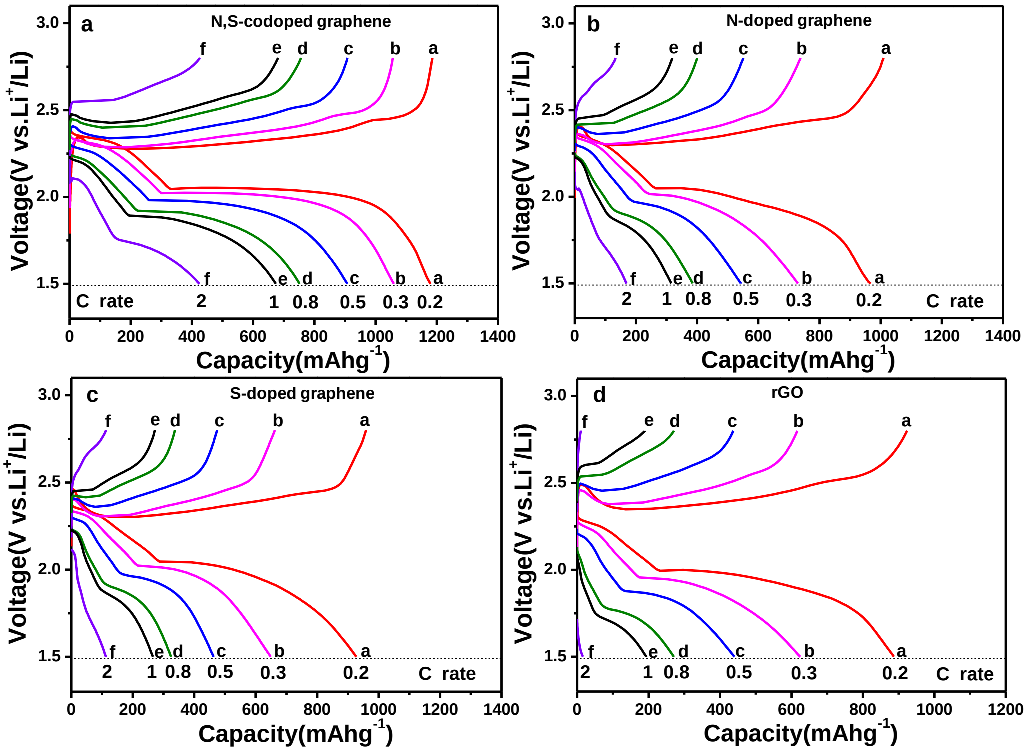

Supplementary Figure 8. Galvanostatic charge-discharge profiles of the (**a**) N,S-codoped graphene, (**b**) N-doped graphene, (**c**) S-doped graphene, and (**d**) rGO electrodes at various rates within the potential window of 1.5 - 2.8 V *vs.* Li^+/Li^0.

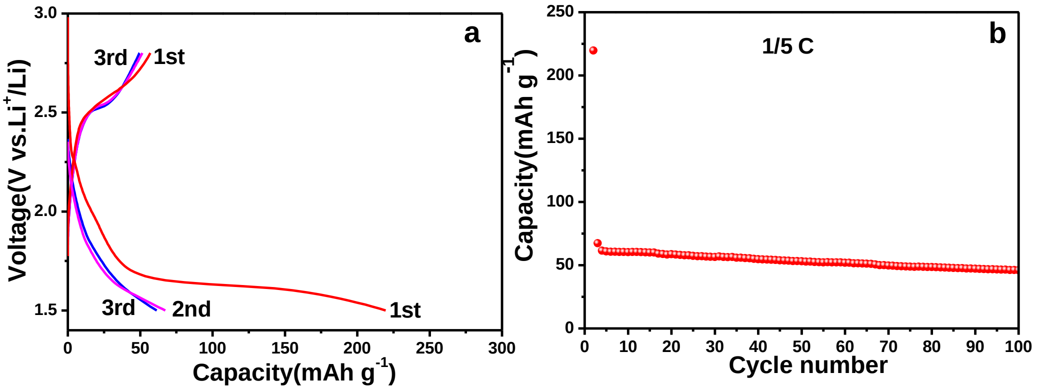

Supplementary Figure 9. (**a**) Blank galvanostatic charge-discharge profiles of the N,S-codoped graphene electrode at 0.2C rate for the first three cycles. (**b**) Cycling stability of the pure N,S-codoped graphene electrode at 0.2C rate for 100 cycles.

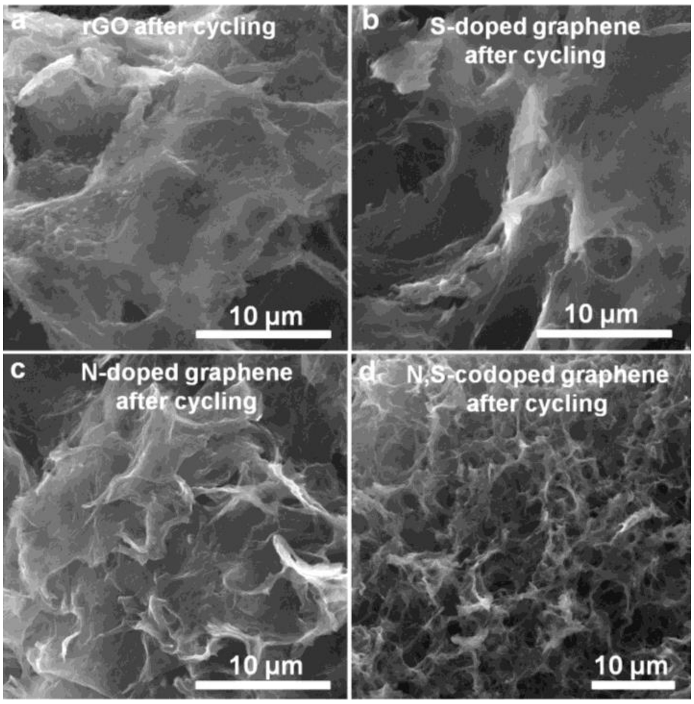

Supplementary Figure 10. SEM images of the (**a**) rGO, (**b**) S-doped graphene, (**c**) N-doped graphene, and (**d**) N,S-codoped graphene electrodes after 100 cycles.

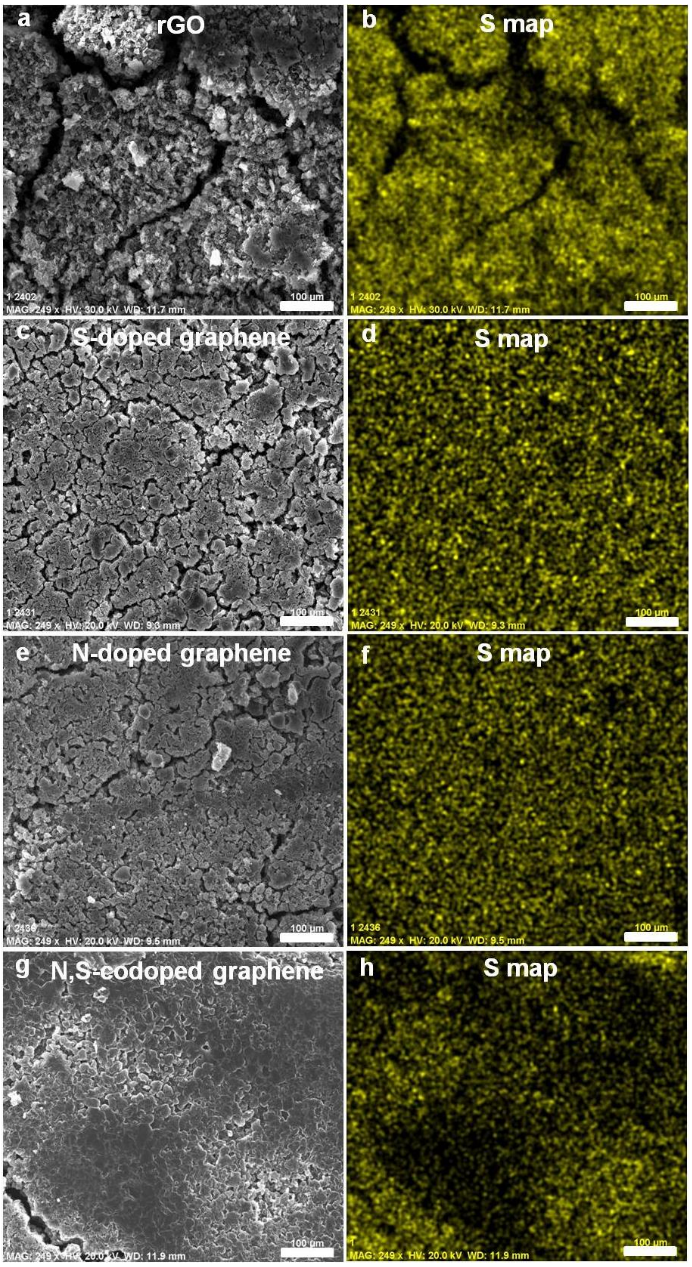

Supplementary Figure 11. SEM images of the lithium-metal surface after 100 cycles in the (**a**) rGO, (**c**) S-doped graphene, (**e**) N-doped graphene, and (**g**) N,S-codoped graphene electrodes. EDS mapping of the lithium anode after 100 cycles in the (**b**) rGO, (**d**) S-doped graphene, (**f**) N-doped graphene, and (**h**) N,S-codoped graphene electrodes. EDS mapping was scanned using the same time for a comparison.

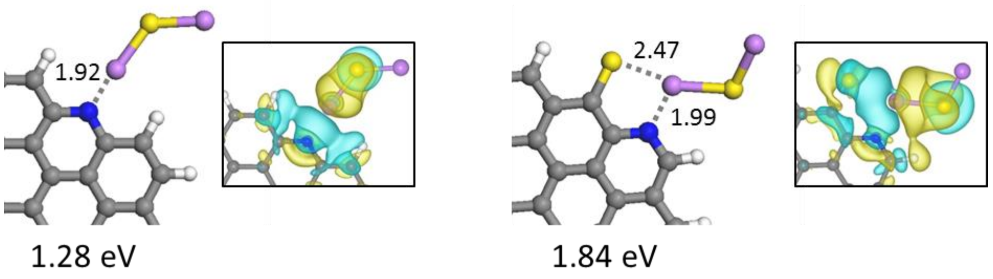

Supplementary Figure 12. Adsorption configurations of Li_2S at two selected dopant sites. The Li_2S binding energies and the distances (in Å) between the Li^+ (in Li_2S) and S/N (in graphene) are also indicated. Charge density difference isosurfaces are shown in the insets; the blue and yellow colors indicate the regions of charge gain and loss (of ±0.001 e/bohr^3), respectively. Gray, white, blue, yellow, and purple balls represent C, H, N, S, and Li atoms, respectively. The comparison with **Fig. 6** [(f) and (i)] clearly shows that there are no significant differences in the binding strength of the terminal Li^+ between Li_2S and LiSH.

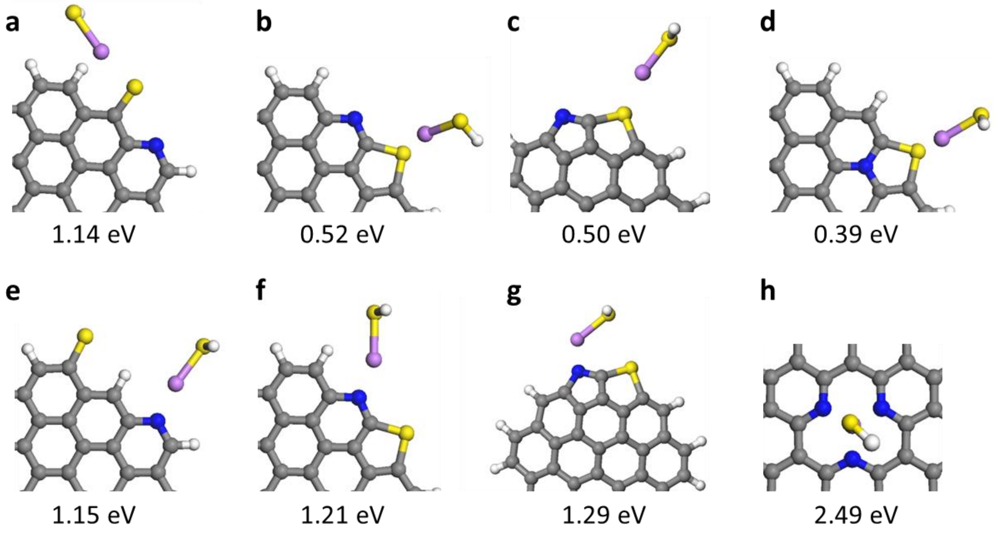

Supplementary Figure 13. Optimized configurations for the binding of LiSH to doped graphene with corresponding LiSH binding energies in eV. Gray, white, blue, yellow, and purple balls represent C, H, N, S, and Li atoms, respectively.

Supplementary Table 1. BET surface area and pore volume of the rGO, S-doped graphene, N-doped graphene, and N,S-codoped graphene

Sample	BET surface area [$m^2\ g^{-1}$]	Total pore volume [$cm^3\ g^{-1}$]
rGO	108.7	0.53
S-doped graphene	133.5	0.60
N-doped graphene	136.7	0.81
N,S-codoped graphene	171.4	1.28

Supplementary Table 2. Elemental contents of C, O, N, S and the ratio between C and O in GO, rGO, S-doped graphene, N-doped graphene, and N,S-codoped graphene sponges

Sample	C(at%)	O(at%)	N(at%)	S(at%)	C/O
GO	70.2	29.8	—	—	2.4
rGO	87.3	12.7	—	—	6.9
S-doped graphene	89.5	9.9	—	0.6	9.0
N-doped graphene	85.9	9.0	5.1	—	9.5
N, S-codoped graphene	83.4	7.3	5.4	3.9	11.4

Supplementary Table 3. Impedance values of the equivalent circuit fitted for the impedance spectra

Sample	R_e (Ω)	R_f (Ω)	R_{ct} (Ω)
rGO	13.7	103.4	469.6
S-doped graphene	5.5	83.5	296.4
N-doped graphene	4.3	72.1	238.7
N, S-codoped graphene	3.7	61.6	147.5

Supplementary Table 4. EDS compositional analysis of the lithium-metal surface after 100 cycles in the rGO, S-doped graphene, N-doped graphene, and N,S-codoped graphene electrodes

Sample / Composition	rGO	S-doped graphene	N-doped graphene	N,S-codoped graphene
Carbon (wt%)	4.8	10.1	13.3	11.8
Nitrogen (wt%)	1.5	2.5	3.5	3.4
Oxygen (wt%)	52.1	54.2	42.6	49.4
Fluorine (wt%)	16.2	14.7	26.7	26.9
Sulfur (wt%)	25.4	18.5	13.9	8.5
